# P-741. Dalbavancin Rationale and Clinical Outcomes in Patients Hospitalized with Serious Bacterial Infections at an Urban Safety Net Hospital: an Observational Cohort

**DOI:** 10.1093/ofid/ofae631.937

**Published:** 2025-01-29

**Authors:** Julieta L Rodriguez, Amanda Roy, Ayesha Ashley Appa, Bruno Bellman, Andrew Banh, Nicholas Campalans, Beatrice Huang, Lisa Gail Winston, John Szumowski, Carina Marquez, Nancy K Hills, Vivek Jain

**Affiliations:** UCSF, San Francisco, California; Zuckerberg San Francisco General Hospital & Trauma Center/UCSF, San Francisco, California; UCSF Infectious Diseases & Addiction Medicine, Mill Valley, California; Zuckerberg San Francisco General Hospital and Trauma Center, San Jose, California; Zuckerberg San Francisco General Hospital and Trauma Center, San Jose, California; UCSF, San Francisco, California; Zuckerberg San Francisco General Hospital, University of California, San Francisco (UCSF), San Francisco, California; UCSF, San Francisco, California; University of California, San Francisco, San Francisco, California; Division of HIV, ID, Global Medicine, University of California San Francisco, Zuckerberg San Francisco General Hospital , San Francisco, CA; UCSF, San Francisco, California; Division of HIV, Infectious Diseases & Global Medicine, San Francisco General Hospital, University of California, San Francisco, San Francisco, CA

## Abstract

**Background:**

Dalbavancin is an emerging therapeutic option for gram positive bacterial infections in patients for whom standard IV or PO therapies are less desirable due to housing instability, substance use, or psychiatric conditions. Data on success rates of dalbavancin, rationale for use, and as step down therapy in at-risk populations are limited. In a large urban cohort of hospitalized patients receiving dalbavancin, we assessed rationale for therapy and clinical outcomes following administration.

Patient demographic and clinical characteristics and rationale for dalbavancin among inpatients receiving therapy (N=107)

**Figure:**
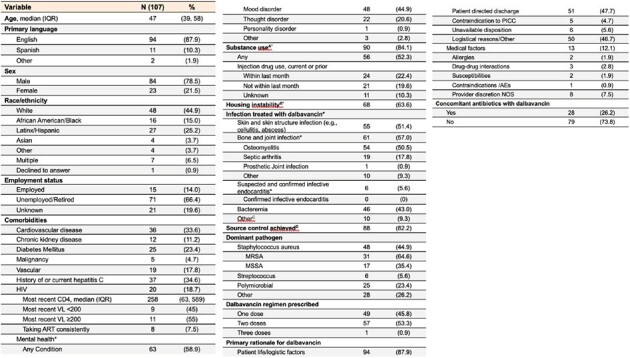

*Patients may have more than one

A: Substance use included: amphetamines including methamphetamine, cocaine, opioids, and other non-prescribed drugs.

B: Housing instability included: any unhoused status including staying on street, in shelter, with friends/family, in vehicle, etc.

C: Other infections included: pneumonia, lymphadenitis, septic thrombophlebitis, septic emboli, septic bursitis, mycotic aneurysm, myositis, TMJ septic arthritis, diabetic foot infection, and unknown.

D: Source control was considered achieved if bacteremia was cleared, incision and drainage was performed, or hardware/prosthetic line/device was removed.

**Methods:**

All adult inpatients who received ≥ 1 dalbavancin doses between 12/2021-3/2024 at Zuckerberg San Francisco General Hospital were evaluated. We assessed patients’ demographic features, provider rationale for dalbavancin use, and treatment completion rate. Time-to-event analysis assessed timing of clinical events. Primary outcome was treatment success (lack of recurrence/nonresolution at 90 days post-dalbavancin by in-depth chart review). Secondary outcomes were 90-day hospital re-admission, dalbavancin completion, and adverse effects.

**Figure:**
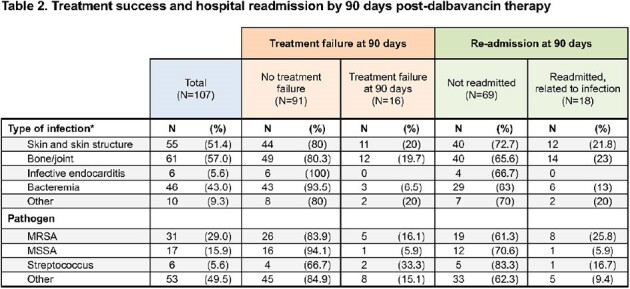

**Results:**

Overall, 107 patients received dalbavancin, and 48% of treatments were in a context of patient self-discharge from hospital. Co-morbidities were prevalent and included HIV (19%; 11/20 [55%] had viral suppression), substance use (84%), psychiatric conditions (59%), and housing instability (64%). Dalbavancin was used to treat skin/skin structure infections (55/107; 51%), bacteremia (46/107; 43%), bone/joint infection (61/107; 57%), and endocarditis (6/107; 6%). Treatment success was seen in 85% of patients, and 88% with *Staphylococcus aureus*. Time-to-event analysis shows clinical failures occurred evenly throughout the 90 days post dalbavancin. Ninety-day hospital readmission was 17%. Adverse events were rare (6%). Dalbavancin dose completion for those prescribed > 1 dalbavancin was 77%. Incomplete therapy had a trend towards lower treatment success (5/13, 39%, P=0.09).

**Figure:**
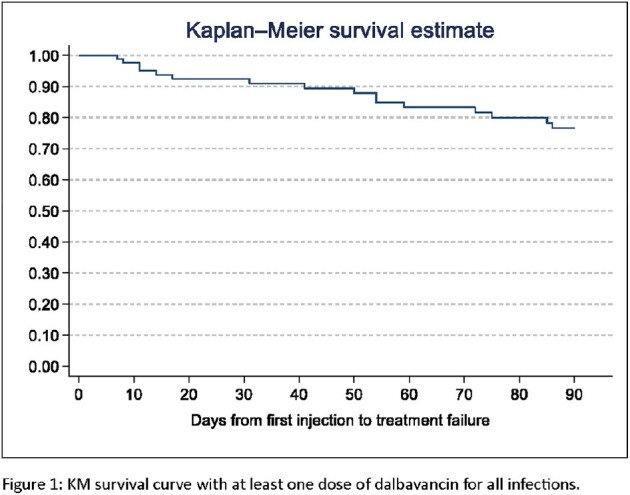

**Conclusion:**

Dalbavancin achieved high treatment success and completion among patients with high prevalence of housing instability, substance use, and medical/psychiatric co-morbidities. Dalbavancin has potential as a patient centered alternative to standard antibiotic therapies in at-risk patients.

**Disclosures:**

**All Authors**: No reported disclosures

